# Absence of genomic hypomethylation or regulation of cytosine-modifying enzymes with aging in male and female mice

**DOI:** 10.1186/s13072-016-0080-6

**Published:** 2016-07-13

**Authors:** Niran Hadad, Dustin R. Masser, Sreemathi Logan, Benjamin Wronowski, Colleen A. Mangold, Nicholas Clark, Laura Otalora, Archana Unnikrishnan, Matthew M. Ford, Cory B. Giles, Jonathan D. Wren, Arlan Richardson, William E. Sonntag, David R. Stanford, Willard Freeman

**Affiliations:** Oklahoma Center for Neuroscience, Oklahoma City, OK USA; Reynolds Oklahoma Center on Aging, SLY-BRC 1370, 975 NE 10th St, Oklahoma City, OK 73104 USA; Department of Physiology, University of Oklahoma Health Sciences Center, Oklahoma City, OK USA; Department of Biochemistry and Molecular Biology, Pennsylvania State University, University Park, PA USA; Division of Neuroscience, Oregon National Primate Research Center, Beaverton, OR USA; Arthritis and Clinical Immunology Program, Oklahoma Medical Research Foundation, Oklahoma City, OK USA; Department of Biochemistry and Molecular Biology, University of Oklahoma Health Sciences Center, Oklahoma City, OK USA; Oklahoma City VA Medical Center, Oklahoma City, OK USA; Department of Geriatric Medicine, University of Oklahoma Health Sciences Center, Oklahoma City, OK USA

**Keywords:** Aging, DNA methylation, DNA hydroxymethylation, TET, DNMT, 5mC, 5hmC, Hippocampus

## Abstract

**Background:**

Changes to the epigenome with aging, and DNA modifications in particular, have been proposed as a central regulator of the aging process, a predictor of mortality, and a contributor to the pathogenesis of age-related diseases. In the central nervous system, control of learning and memory, neurogenesis, and plasticity require changes in cytosine methylation and hydroxymethylation. Although genome-wide decreases in methylation with aging are often reported as scientific dogma, primary research reports describe decreases, increases, or lack of change in methylation and hydroxymethylation and their principle regulators, DNA methyltransferases and ten-eleven translocation dioxygenases in the hippocampus. Furthermore, existing data are limited to only male animals.

**Results:**

Through examination of the hippocampus in young, adult, and old male and female mice by antibody-based, pyrosequencing, and whole-genome oxidative bisulfite sequencing methods, we provide compelling evidence that contradicts the genomic hypomethylation theory of aging. We also demonstrate that expression of DNA methyltransferases and ten-eleven translocation dioxygenases is not differentially regulated with aging or between the sexes, including the proposed cognitive aging regulator DNMT3a2. Using oxidative bisulfite sequencing that discriminates methylation from hydroxymethylation and by cytosine (CG and non-CG) context, we observe sex differences in average CG methylation and hydroxymethylation of the X chromosome, and small age-related differences in hydroxymethylation of CG island shores and shelves, and methylation of promoter regions.

**Conclusion:**

These findings clarify a long-standing misconception of the epigenomic response to aging and demonstrate the need for studies of base-specific methylation and hydroxymethylation with aging in both sexes.

**Electronic supplementary material:**

The online version of this article (doi:10.1186/s13072-016-0080-6) contains supplementary material, which is available to authorized users.

## Background

Epigenetic control of the genome through DNA and histone modifications is potentially a critical regulator of the aging process [1]. For DNA methylation in particular, recent studies have revealed the possibility of an aging “clock” in the form of DNA methylation that indicates chronological age [2, 3]. DNA methylation changes with aging may even be predictive of mortality [4]. In the nervous system, DNA methylation [5] and hydroxymethylation [6] have emerged as critical processes in memory formation [7] that may be disrupted with aging, leading to cognitive decline [8, 9]. Altered epigenome regulation is also potentially a contributor to age-related neurodegeneration including Alzheimer’s disease [10]. From a geroscience perspective [11], it is essential that we clarify and expand our understanding of aging-associated alterations of epigenetic processes in the brain to determine their contribution to health and disease.

DNA methylation (mC) and hydroxymethylation (hmC) occur at the 5 position of the pyrimidine ring of cytosines, and while predominantly occurring in CG dinucleotide contexts, it is also found in CH (H being C, A, or T) contexts [12]. DNA methyltransferases (DNMTs) maintain methylation patterns during cell division (DNMT1) and add de novo mC (DNMT3a and DNMT3b) in response to stimuli [13]. Tet methylcytosine dioxygenases (TETs) oxidize mC into hmC, as well as the rarer formylcytosine (fC) and carboxylcytosine (caC) [14]. Several hypotheses regarding DNA methylation with brain aging have been proposed. These include (1) genomic hypomethylation, a genome-wide decrease in methylation levels across the genome [15]; (2) regulated changes, both hypomethylation and hypermethylation, at specific genomic locations, that are unique to individual cells and organs [16]; (3) an aging clock of regulated changes in methylation at common locations across tissues/cells and even species [3]; and (4) epigenetic drift where DNA methylation patterns become disordered and diverge with time [17].

It is often stated that there is less global genomic methylation (hypomethylation) with aging as a consequence of decreased DNMT expression, and this is often presented as dogma in review articles [15, 18–20]. However, examination of the primary literature regarding hippocampal aging presents contradictory evidence for both mC levels [21–25] (Additional file 1: Table S1) and DNMT expression [8, 21, 25–27] (Additional file 1: Table S2). In studies of wild-type rodent laboratory models and aging time points that meet well-established definitions [28], increased, decreased, and no change in total mC and DNMT expression have been reported. DNMT3b is an exception with no reports of changes in expression with aging. The discordance in the literature is not obviously attributable to the models used (e.g., by mouse strain) or the endpoints assessed (e.g., mRNA vs protein). Reports on hmC (Additional file 1: Table S3) and its regulating TET genes (Additional file 1: Table S4) are less numerous, but also do not present a clear understanding of their regulation with aging [22, 29–31]. A notable omission evident in this literature is the absence of studies in female animal models. Given the profound sex differences in brain aging [32–35], sex-specific regulation of DNA methylation (e.g., X-inactivation [36]), and the laudable recognition that both sexes need to be analyzed separately in preclinical research [37], studies examining both sexes with aging are clearly warranted.

To address this fundamental lack of clarity in the literature, genomic mC and hmC and the expression of the genes responsible for these modifications, DNMTs and TETs, respectively, were assessed in the hippocampus of young (3 months), adult (12 months) and old (24 months) male and female C57Bl6 mice using a variety of analytical methods. Our results demonstrate: (1) absence of a change in total genomic levels of 5mC or 5hmC and expression of DNMTs and TETs with aging and (2) DNMT3a2, a proposed regulator of age-related cognitive decline [8], not expressed at physiologically relevant levels after early development. These data resolve several critical controversies of epigenetic changes with brain aging. We find that sex- and age-related differences in average mC and hmC occur within specific cytosine contexts (CG/CH), X chromosome, and genomic elements such CG island (CGI) shores and shelves, and in promoter regions. These findings demonstrate the need for studies examining age-related mC and hmC changes in a base- and strand-specific manner across the genome in both sexes to identify specific regions of differential modification with aging. Future studies of specific, isolated cell types are also needed to extend these findings. The study also demonstrates the limitations of many commonly used epigenomic methods and provides approaches for quantitatively rigorous epigenomic studies through replication and inclusion of quantitative controls.

## Results

**Fig. 1:**
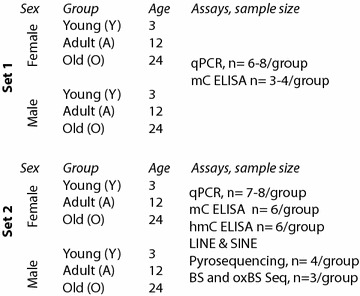
Experimental design. Design of the study including animal groups, assays performed, and sample sizes for each assay. For both sets of mice, total sample size was *n* = 8–10/sex–age

Two independent cohorts of young (3 M), adult (12 M), and old (24 M) male and female C57Bl6 mice were generated for these studies (Fig. 1). Mice were purchased from the NIA Aging Rodent Colony and housed at two independent institutions where they were allowed to acclimate. Female mice were monitored for estrous cycle stage by vaginal lavage and microscopy. At tissue collection weight were 3 M male (27.7 ± 1.5), 3 M female (21.7 ± 1.1), 12 M male (30.8 ± 1.7), 12 M female (24.1 ± 1.4), 24 M male (32.1 ± 1.9), 24 M female (27.2 ± 1.5). Mice were euthanized and hippocampi dissected and flash frozen, with all females being in diestrus at the time of tissue collection. Both sets of animals were used for the gene expression studies. mC ELISA analysis was also performed on both cohorts. After exhaustion of the DNA samples in Set 1 (much of the sample being used in other studies), the remainder of the DNA analyses (pyrosequencing, hmC ELISA, and oxidative bisulfite sequencing) were performed on Set 2.

### DNMT expression and aging

**Fig. 2:**
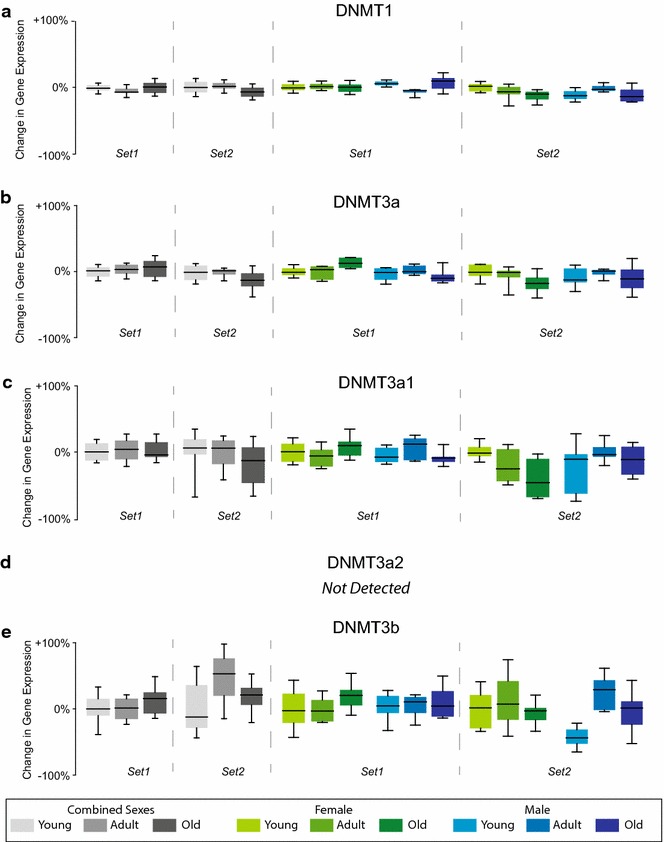
Gene expression analysis of DNMTs. mRNA levels of DNMTs were assessed by qPCR in hippocampi of both sets of mice. Data were analyzed both with sexes combined (one-way ANOVA on age) and with sexes analyzed independently (two-way ANOVA on age and sex). No consistent changes were evident in **a** DNMT1, **b** pan-DNMT3a (combination of long, a1, and short, a2, isoforms), **c** DNMT3a1, or **e** DNMT3b. **d** DNMT3a2 was not detectable. Data are scaled to zero for the young group in the combined sexes analysis and to young female for the sex separated analysis with the *y*-*axis* scale presented as % change from zero. *Boxes* encompass the 25th to 75th percentile with *bars* indicating 10th and 90th percentiles. *Median lines* are indicated in the *boxes*

To first examine mRNA expression of DNMTs, qPCR was performed on both mouse cohorts. DNMT1, DNMT3a and DNMT3b were examined (Fig. 2). For DNMT3a, three independent assays were used, as DNMT3a is expressed as both a long (DNMT3a1) and short form (DNMT3a2) across mammalian species [38]. Assays were designed to detect a common region of DNMT3a1 and DNMT3a2 (DNMT3a), as well as isoform-specific regions of DNMT3a1 or DNMT3a2. No consistent age-related changes [two-way ANOVA with sex and age as factors and Benjamini–Hochberg multiple testing correction (BHMTC) for the number of genes examined] across the two cohorts in any of the DNMT mRNAs examined were evident, regardless of whether the analysis combined both sexes or was analyzed in a sex-specific manner (Fig. 2). No sex differences were observed in expression of any of the mRNAs. DNMT3a2 was not detected in the qPCR experiments in either animal cohort.

### DNMT3a2 developmental expression and with aging

**Fig. 3:**
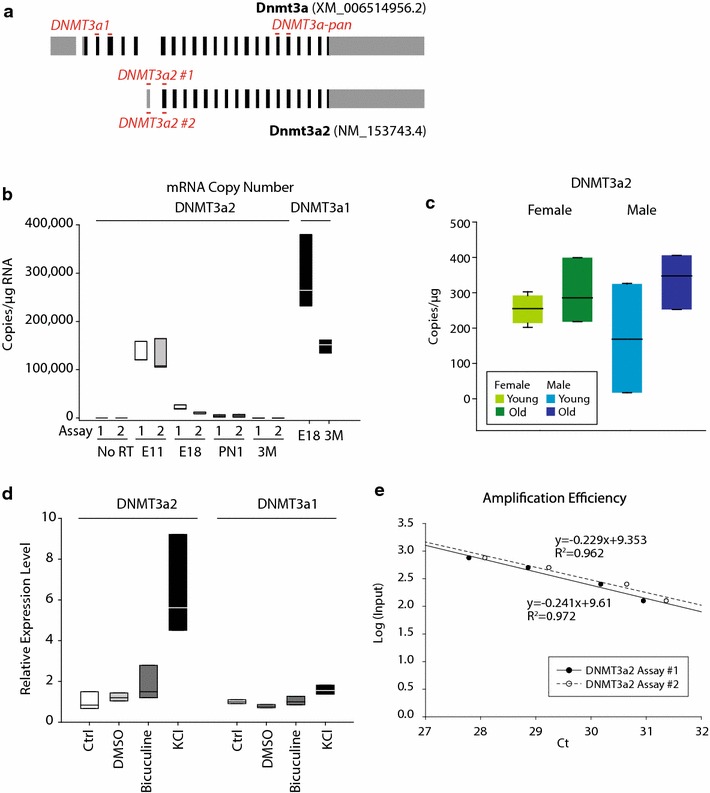
DNMT3a2 mRNA expression. Four independent sets of gene expression assays were used in these studies to specifically examine (**a**) long (DNMT3a1), short (DNMT3a2), or both isoforms. DNMT3a2 copy number, expressed as copy number per microgram of RNA, was analyzed (**b**) in E11, E18, and PN1 brain and in mature (3 M) hippocampus (HP). Expression declined from a peak level at E11 to a low at 3 M that was slightly higher than background levels (No RT). DNMT3a1 copy number was analyzed for comparison in E18 brain and 3 M old hippocampus. **c** DNMT3a2 copy number was analyzed with Assay #1 in hippocampi from young and old, male and female Set 2 mice (*n* = 4/group). **d** Induction of DNMT3a2 by KCl, and to a lesser extent bicuculline in hippocampal embryonic cultures was determined by qPCR. **e** Amplification efficiency of both DNMT3a2 assays was derived from a dilution series of input cDNA and in both cases was found to be 75 %

The lack of DNMT3a2 expression was unexpected considering the assay used (Assay #1, Additional file 1: Table S5) is identical to the one used in a previous report that describes a decline in expression in the mature hippocampus with aging [8]. DNMT3a2 is a truncated form of DNMT3a found across mammals that utilizes an alternate transcription start site [38, 39]. While DNMT3a2 does not have any unique protein-coding sequence, it has an alternate 5′ UTR which allows specific detection of this mRNA. To further examine DNMT3a2, we designed a second assay (Assay #2) shifted slightly from the location of the primers and probe for Assay #1 to specifically detect DNMT3a2 (Fig. 3a). No DNMT3a2 expression was reliably observed by qPCR in either Set 1 or Set 2 mice with Assay #2 as well. To ensure that both assays were amplifying the appropriate sequence, amplicons were generated from embryonic brain (E11) RNA, which contains high levels of DNMT3a2 [40]. Amplicons were examined by electrophoresis and Sanger sequencing, and both assays were found to produce only the desired amplicon of the appropriate sequence (data not shown).

While these data suggest that DMNT3a2 is not expressed in the adult hippocampus, it could be that the qPCR approach is not sufficiently sensitive to detect very low levels of DNMT3a2 expression. To increase sensitivity, both assays were used in a chip-based digital PCR format which contains 20,000 individual wells to absolutely quantify/count the number of transcript copies in embryonic brain (E11 and E18), postnatal brain (PN1), and mature hippocampus (3 M) without the need for endogenous qPCR controls that often change in expression over development. DNMT3a2 has been reported to be highly expressed during early development in the CNS and then declines over time with no expression evident in adults [40, 41]. DNMT3a2 transcript levels were highest at E11, decreasing by an order of magnitude at each step to E18, PN1, and mature hippocampus (3 M) as measured by both assays (Fig. 3b). Expression levels were at the limit of detection for the mature hippocampus (~200 copies/µg RNA), with background levels, as determined by no reverse transcription controls, being ~15 copies/µg RNA. For comparison, levels of DNMT3a1 were examined in E18 brain and mature hippocampus. At E18, brain DNMT3a1 levels were tenfold higher than DNMT3a2, and in mature mice, hippocampal DNMT3a1 levels were nearly 1000-fold higher than DNMT3a2. To address the possibility that the previously reported changes in DNMT3a2 expression with aging are in these near background levels of expression, the dPCR approach was applied to young and old male and female mouse hippocampal samples. No difference in these very low levels (~200 copies/ug of RNA) of DNMT3a2 was observed (Fig. 3c).

Previous reports have described an induction of DNMT3a2 expression with bicuculline or KCl treatment in embryonic hippocampal cultures [8], and replication of these findings was also attempted as a positive control. Repeating these experiments, inductions in DNMT3a2 expression, but to a smaller extent than previously reported, were evident in cultures (Fig. 3d). Seeking to identify a potential source of the differences between the findings of this study and previous reports, the amplification efficiency of the DNMT3a2 assays was examined. Efficiencies were found to be 76 % for Assay #1 and 75 % for Assay #2 (Fig. 3e). Previous quantitation assumed 100 % amplification efficiency (calculating group differences as $2^{{{\Delta \Delta }C_{\text{t}} }}$ instead of $1.75^{{{\Delta \Delta }C_{\text{t}} }}$) and would therefore overestimate differences between groups. This potentially provides a reason that no changes were observed with aging and the inductions with bicuculline and KCl were not as large.

**Fig. 4:**
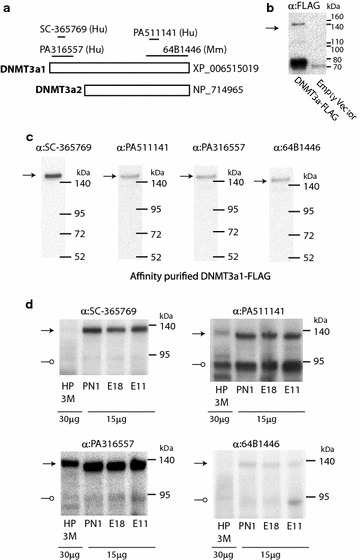
DNMT3a2 protein expression. **a** Four different antibodies were used in these experiments, two with antigens in the DNMT3a1-specific N-terminal region and two with antigens in the DNMT3a1/DNMT3a2 common C-terminal region. **b** As a positive control and to provide a reference molecular weight, the full-length human DNMT3a protein with a FLAG tag was overexpressed in HEK cells. Cell lysates with the overexpression vector and empty vector were probed with anti-FLAG antibody. DNMT3a was detected at ~140 kDa (*arrow*), but a non-specific band at ~75 kDa was also detected. Therefore, the FLAG-tagged DNMT3a was affinity purified on an anti-FLAG column. **c** The purified protein (0.1 μg) was probed with all four primary antibodies, returning a single band at ~140 kDa (*arrows*) demonstrating that all four antibodies could detect DNMT3a at the correct molecular size for future experiments. **d** Protein lysates from E11, E18, PN1 brain and 3 M hippocampus (15 μg) were probed with all four primary antibodies. The full-length DNMT3a was detected with all antibodies though with very weak signal in 3 M hippocampal samples (data not shown). To increase the likelihood of detecting DNMT3a in the hippocampal sample, immunoblots were performed again but with twice the protein loading in hippocampal samples (30 μg) as the other samples (15 μg). Full-length DNMT3a was detected including in hippocampal samples (*arrows*)—though the signal was very low for SC-36769 and 64B1446 antibodies. As the C-terminal antibodies should also detect the shorter form of DNMT3a, DNMT3a2, the PA511141 and 64B1446 blots were examined for banding not found with the N-terminal antibodies. While a ~95-kDa band was observed, it was also evident with the N-terminal antibodies (*open circles*). No unambiguous banding was evident that could be attributed to DNMT3a2

Though previous reports [8] examining DNMT3a2 expression with aging did not find protein expression in the old mouse hippocampus, the possibility remains that DNMT3a2 expression could be much higher at the protein than mRNA level. Given that the literature reports many different molecular weights for DNMT3a1 and DNMT3a2 [38, 42, 43] and the high levels of homology with DNMT3b, a set of polyclonal and monoclonal antibodies against the DNMT3a1-specific N-terminus and DNMT3a1/DNMT3a2 common C-terminus (Fig. 4a) including those in previous reports [8, 38, 44] were used to detect DNMT3a. First a FLAG-tagged DNMT3a1 was overexpressed in HEK cells to provide a positive control (Fig. 4b). DNMT3a1-FLAG was affinity purified, and purified protein was probed with all four primary antibodies (Fig. 4c) giving a single band at ~140 kDa providing evidence that all four antibodies can detect DNMT3a1. Protein lysates from E11, E18, and PN1 brain and 3 M hippocampus were probed with all four antibodies. DNMT3a1 was initially detected in E11, E18 and PN1 samples, but not in mature hippocampus (data not shown). Samples were rerun with twice the protein load (30 μg) for the hippocampus as the E11, E18, and PN1 brain (15 μg) (Fig. 4d). The full-length DNMT3a1 was detected in all samples, though at a lower level in the hippocampus even given the high protein loading. To identify DNMT3a2, banding was examined for species present in the C-terminal antibodies, but not present in the N-terminal antibodies. DNMT3a2 is predicted to be ~24 kDa smaller than DNMT3a1. While a major band was evident at ~95 kDa, this was also evident in N-terminal, DNMT3a1-specific antibodies. Some very minor bands were present, but there was no unambiguous identification of DNMT3a2 in any of the samples. While these findings do not obviate the potential for DMNT3a2 protein to be expressed in these samples, the lack of any previous reports definitively demonstrating endogenous DNMT3a2 protein expression in the CNS and the much lower gene expression of DNMT3a2 as compared to DMNT3a1 (above) suggests that DNMT3a1 is the predominant protein form of DNMT3a expressed in the CNS. As previous reports examining DMNT3a2 report many immunoreactive bands [8, 38] and the high levels of C-terminal homology with DNMT3b isoforms [38], development and validation of affinity reagents for detection of DNMT3a2 are needed to definitively answer the question of DNMT3a2 protein expression with aging in the CNS.

### TET expression across sexes and ages

**Fig. 5:**
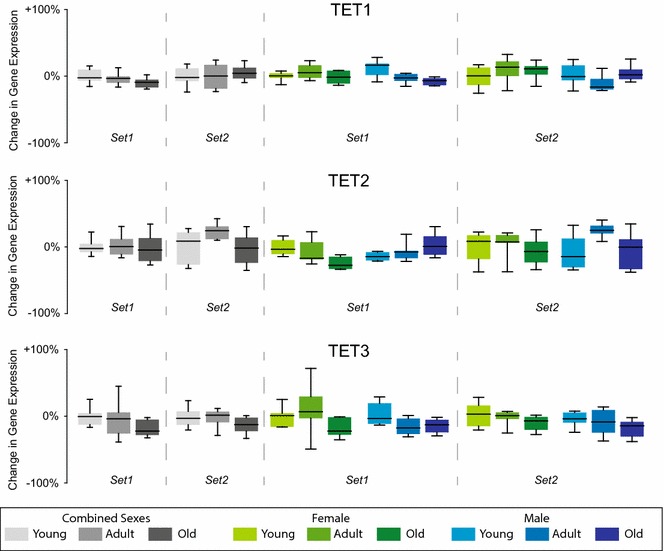
Gene expression analysis of TETs. mRNA levels of TETs were assessed by qPCR in hippocampi of both, sets of mice. Data were analyzed both with sexes combined (One-way ANOVA on age) and with sexes analyzed independently (two-way ANOVA on age and sex). No consistent changes were evident in **a** TET1, **b** TET2, or **c** TET3. Data are scaled to zero for the young group in the combined sexes analysis and to young female for the sex separated analysis with the *y*-*axis* scale presented as % change from zero. *Boxes* encompass the 25th to 75th percentile with *bars* indicating 10th and 90th percentiles. *Median lines* are indicated in the *boxes*

For completeness, the expression of TET1, TET2, and TET3 was examined in both cohorts of mice. No consistent age- or sex-related changes (two-way ANOVA on sex and age with BHMTC) in the hippocampal expression of any of the TET genes were observed (Fig. 5).

### Meta-analysis of public data for DNMT and TET expression

Utilizing public human gene expression data from the NCBI Gene Expression Omnibus for which age and tissue data could be extracted, a linear mixed-effects model was constructed to estimate DNMT and TET expression with age. Analyzing the general signature of aging across all available tissues for 16,044 samples suggested no evident correlation of gene expression with age (Additional file 1: Table S7). Analysis of 496 samples annotated as “brain” (including specific substructures) (Table 1) similarly showed no significant changes in expression with age.

**Table 1:** Meta-analysis of age-related DNMT and TET expression in brain

Entrez ID	Symbol	Chr	Name	Coeff.	SE	*t*	*p* value
80312	TET1	10	Tet methylcytosine dioxygenase 1	−0.00192	0.006	−0.34	0.73
54790	TET2	4	Tet methylcytosine dioxygenase 2	−0.00002	0.005	−0.004	1.0
200424	TET3	2	Tet methylcytosine dioxygenase 3	−0.00033	0.005	−0.064	0.95
1786	DNMT1	19	DNA (cytosine-5-)-methyltransferase 1	0.00784	0.007	1.093	0.27
1788	DNMT3a	2	DNA (cytosine-5-)-methyltransferase 3 alpha	0.004	0.004	0.980	0.33
1789	DNMT3b	20	DNA (cytosine-5-)-methyltransferase 1	−0.00009	0.004	-0.02	0.98

To test whether the expression levels of these genes change with age, we used a linear mixed-effects (lme4) model: [GeneExpression ~ Age: TissueID + (1|ExperimentID) + (1|PlatformID)]. Shown above is the age–tissue interaction term for brain. “Coef” describes the estimated coefficient of the change in *Z*-score per year for the expression of each gene, the standard error (SE), and *t*-statistic for the analysis. The *p* value shows that none of the estimated gene expression changes with age are statistically significant

### Methylcytosine (mC) levels with aging and between sexes

**Fig. 6:**
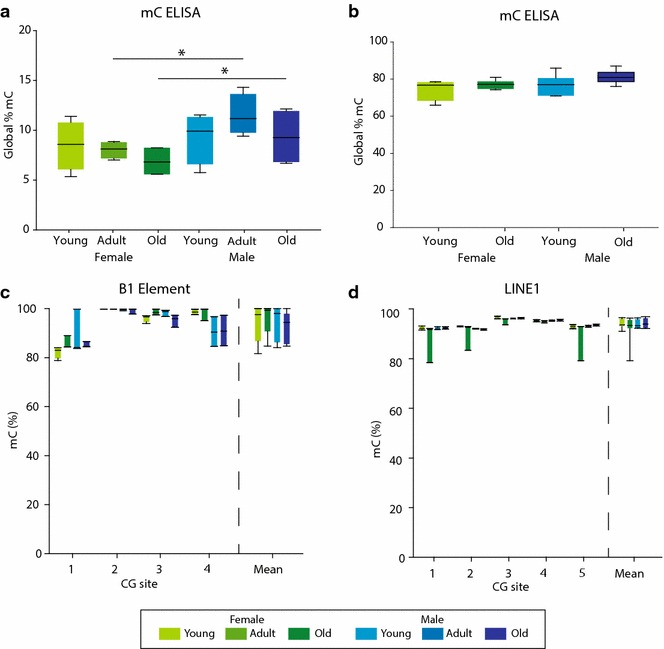
Global mC levels: ELISA and pyrosequencing. **a** Total/global levels of hippocampal mC were analyzed by ELISA with synthetic methylation standards (young, adult, and old, females and males Set 1). (*p < 0.05 Student–Newman–Keuls pairwise post hoc test following two-way ANOVA). **b** Using a mouse whole-genome methylation standard curve, the Set 2 samples (young and old, females and males) were analyzed by mC ELISA. Repeat element methylation was analyzed by pyrosequencing of the **c** B1 element and the **d** LINE1 repeat (young and old, females and males Set 2). No differences were evident in any of the individual CG sites or in the mean of the element

As the primary literature includes a number of contradictory findings regarding total hippocampal mC levels (Additional file 1: Table S1) with aging and no previous reports have examined female mice, total levels of mC were first analyzed by ELISA. ELISA-based approaches are specific to mC; however, they do not differentiate between CG and CH contexts or provide chromosomal or other locational information on where mC residues are located. Historically, mCG levels have been a focus of attention, but recent findings have conclusively demonstrated non-CG methylation (mCH) is abundant, especially in the CNS [41, 45], and serves a functional role in genomic regulation [46, 47]. No age-related changes were observed (Fig. 6a) in mC, but there was a sex effect (*p* = 0.002 two-way ANOVA, *n* = 4/group) that was evident in pairwise post hoc comparisons [Student–Newman–Keuls (SNK)] for adult (*p* = 0.014) and aged (*p* = 0.038) mice with higher levels in males. Of note was that the observed mC levels of ~10 % were lower than those reported in some ELISA studies [24, 25] and from deep sequencing [41, 45]. The standard curve used with most ELISAs is generated from a small in vitro methylated sequence (mCG only). This sequence has five times the density of CG sites as the mouse genome and therefore likely underestimated the absolute levels in these mouse samples. To more accurately quantify whole-genome levels, mouse methylation standards were generated in which mouse genomic DNA was methylated in vitro by M. SssI to produce a high standard (~95 % mCG) or whole genome amplified in the absence of methyltransferases to produce a low methylation standard (<5 % mCG). Both high and low standards were verified by pyrosequencing. The ELISA was then repeated with these standards, and DNA from only young and old Set 2 mice was analyzed to increase sample size (*n* = 6/group). With the appropriate standards, the absolute levels of mC were similar to those reported in sequencing studies. Again, no age effect was observed, and a marginally higher level of total mC was evident in males versus females (*p* < 0.05, factor of sex, two-way ANOVA) (Fig. 6b). However, no significant pairwise post hoc differences (SNK) were observed.

A proposed surrogate endpoint for total methylation levels is to examine repeat element methylation levels by pyrosequencing [48, 49]. This has the advantage of specifically examining repeat elements such as long interspersed repeat elements (LINEs) and short interspersed repeat elements (SINEs), which account for 19 and 8 % of the genome, respectively [50], with the caveats that bisulfite conversion cannot distinguish between mC and hmC. The SINE element B1 (Fig. 6c) and the LINE element LINE1 (Fig. 6d) were examined both at the individual CG site level and by combining the methylation levels from all the sites in the element. Repeat elements were highly methylated, approaching 100 %. No effect of age or sex was observed (two-way ANOVA), and methylation levels approached 100 % in the repeat elements.

### Total hmC levels

**Fig. 7:**
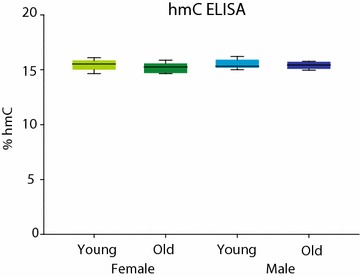
Global hmC levels: ELISA. Total/global levels of hmC were analyzed by ELISA (young and old, females and males Set 2) with the provided synthetic hydroxymethylation standards. No age or sex differences in total hmC were observed

hmC is more abundant in the brain [29, 51] as compared to other tissues. Previous studies have described increased total hmC levels with aging (Additional file 1: Table S3). Total hmC levels were measured by ELISA in young and old, male, and female Set 2 mice (*n* = 6/group). No differences were observed with age or sex (two-way ANOVA) (Fig. 7).

### Whole-genome oxidative bisulfite sequencing

Arguably the most accurate and specific method to quantify mC and hmC levels that also include genomic location and context (CG vs CH) information is whole-genome sequencing. Two methods for base-specific quantitation of mC and hmC have been recently described: TET-assisted bisulfite sequencing (TAB-Seq) [52] and oxidative bisulfite sequencing (oxBS-Seq) [53, 54]. We applied the oxBS-Seq approach to specifically quantifying mC and hmC genome wide in young and old male and female mouse hippocampus. The primary limitation of whole-genome sequencing of mC and hmC is that to quantify every base with strand specificity enormous amounts of sequencing data are required [at least 166 Gb per sample (3.2 Gb genome × 2 strands × 2 libraries × 10× coverage/~70 % of reads passing QC and alignment)]. This is far beyond the scope of this study. However, to take advantage of oxBS-Seq we sequenced genomes at a lower coverage and summed methylation or hydroxymethylation counts for each sample from aligned reads by chromosome and by genomic element [CpG Islands (CGIs), or promoters]. This approach provides hundreds of thousands to hundreds of millions of individual base counts for each sample, and quantitation can be split into different contexts (CG or CH) (Additional file 1: Table S8).

**Fig. 8:**
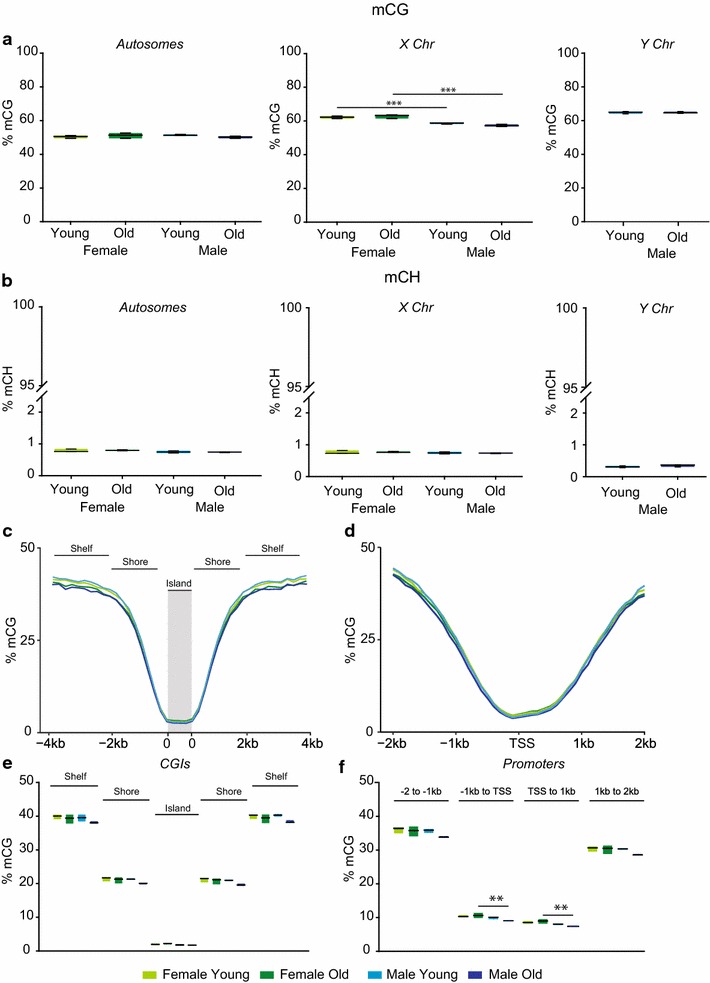
Global mC levels: whole-genome sequencing. Specific levels of mC were analyzed by oxidative bisulfite sequencing. **a** mC levels in the CG context for autosomes, X, and Y chromosomes were analyzed in young and old females and males. **b** In the CH context, mC levels were much lower as compared to CG. **c** mCG levels in CpG islands, shores, and shelves for all annotated CGIs per sample were combined and plotted in 200 bp bins. **d** All annotated promoter regions (±2 kb of TSS, 100-bp bins) methylation was lowest at the TSS with higher levels further upstream and downstream. **e** Statistical comparisons of islands as well as upstream and downstream shores and shelves revealed slightly lower levels in Old males. **f** For promoter regions, mCG levels in 1-kb bins upstream and downstream of the TSS were compared. Two-way ANOVA, SNK post hoc **p < 0.01

Young and old, male and female hippocampus samples (*n* = 3/group) were analyzed by oxBS-Seq. Female samples were aligned to the mouse genome without the Y chromosome, while males were aligned to the complete mouse genome. Methylation counts were separated by CG and CH contexts and by chromosome. No age or sex differences (Two-way ANOVA) in mCG-specific methylation levels were observed for autosomes, but higher X chromosome methylation was evident in females as compared to males (factor of sex *p* = 0.0008) (Fig. 8a) and between young females and males (SNK, *p* = 0.001) and between old females and old males (SNK, *p* = 0.008). There was no difference in male Y chromosome mCG with aging.

Methylation of the CH context (Fig. 8b) was much lower than CGs, as has been previously reported [45]. It should be noted that the number of CH sites in the genome is ten times that of CG sites, and while most CH sites are unmethylated, individual sites can be quite highly methylated (e.g., [55]), thus explaining the low mean values. No sex or age differences were evident in autosomes or the X and Y chromosomes.

Summing the counts within chromosomes does not differentiate between genomic elements which can have different methylation levels and patterns. Methylation counts were averaged across all annotated CGIs and all promoter regions of annotated RefSeq genes for each mouse in 100-bp bins for promoters and 200-bp bins for CGIs (Fig. 8c, d). mCG levels reached nadirs over the islands and at the TSS with higher levels upstream and downstream. This pattern of methylation, low over islands, and near the TSS has been previously observed with traditional bisulfite sequencing of brain tissues, but the absolute values here are lower as the quantitation presented here is mC specific [41]. mCG levels were lower in old males than young males across CGI shores and shelves (Fig. 8e). There were no significant age or sex differences in CGI shores, shelves, or the island itself (two-way ANOVA, BHMTC for multiple regions). In promoter regions, mCG methylation was lower in old males as compared to old females in the 1-kb upstream (two-way ANOVA factor of age, *p* = 0.005, SNK old female versus old male *p* = 0.004) and downstream of the TSS (two-way ANOVA factor of age, *p* = 0.003, SNK old female versus old male *p* = 0.003) (Fig. 8f). These differences were of a small magnitude (<2 % absolute difference).

**Fig. 9:**
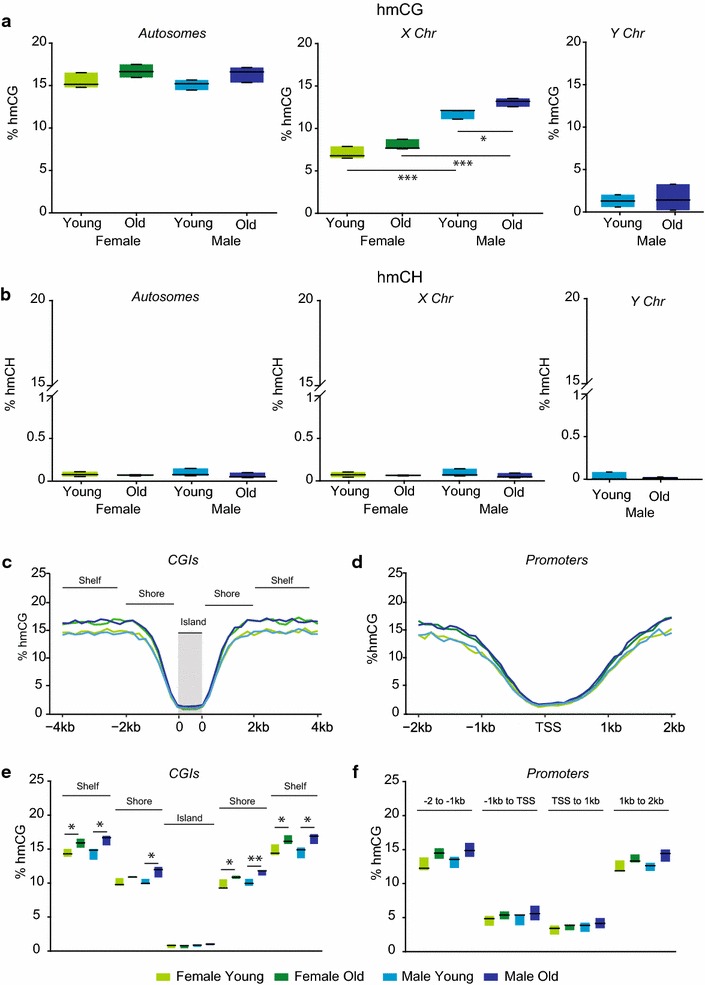
Global hmC levels: whole-genome sequencing. **a** Specific levels of hmC, derived from oxidative bisulfite sequencing, were analyzed for autosomes and sex chromosomes. **b** hmC in the CH context was quantified in autosomes and sex chromosomes. **c** hmCG levels in CpG islands, shores, and shelves for all annotated CGIs per sample were combined and plotted in 200-bp bins. **d** In promoter regions (±2 kb of TSS, 100-bp bins), hydroxymethylation was lowest around the TSS with higher levels further upstream and downstream. **e** Statistical comparisons of islands as well as upstream and downstream shores and shelves revealed higher hmCG levels in shores and shelves with aging. **f** In promoter regions, hmCG levels were greater in 1- to 2-kb bins upstream in females with aging and 1- to 2-kb downstream of the TSS in males with aging. Two-way ANOVA, SNK post hoc *p < 0.05, **p < 0.01

For hmC quantitation in CG contexts, there were no significant differences in autosomal hmCG (Fig. 9a). On the X chromosome, hmCG methylation was greater in males than in females as well as in old males as compared to young males (two-way ANOVA factor of sex *p* = 0.0002, SNK young female versus young male *p* = 0.007, SNK old female versus old male *p* = 0.0005). There was no difference in hmCG levels with aging on the Y chromosome, and methylation levels were much lower than other chromosomes. In the CH context, hmC levels were extremely low (<0.1 %), consistent with previous reports of other CNS regions [56] (Fig. 9b), and no differences with sex or age were observed. In CGIs and promoter regions, hmCG levels were lowest in the island itself and around the TSS before reaching near genome-wide levels (~15 %) upstream and downstream (Fig. 9c, d). In CGIs (Fig. 9e), a higher level of hmCG in shelves (Two-way ANOVA, BHMTC, factor of age *p* = 0.003, SNK female young versus old *p* = 0.036, SNK male young versus old *p* = 0.01) and shores (two-way ANOVA, BHMTC, factor of age *p* = 0.004, SNK male young versus old *p* = 0.007) upstream of the island with aging were observed. A similar pattern in the downstream shelves (Two-way ANOVA, BHMTC, factor of age *p* = 0.004, SNK female young versus old *p* = 0.038, SNK male young versus old *p* = 0.013) and shores (two-way ANOVA, BHMTC, factor of age *p* = 0.002, SNK female young versus old *p* = 0.022, SNK male young versus old *p* = 0.006) was evident. In promoter regions (Fig. 9f), no sex or age effects passed multiple testing corrections. Similarly to mCG the differences in hmCG with aging were of a small absolute magnitude (<5 %).

## Discussion

Through the application of multiple analytical approaches to independent cohorts of young (3 M), adult (12 M) and old (24 M) male and female mice and using the hippocampus as a sentinel brain structure, we demonstrate that: (1) expression of DNMTs and TETs is not regulated by aging or by sex; (2) DNMT3a2 expression in the mature hippocampus is minimal and does not change with aging; and (3) total genomic methylation and hydroxymethylation levels do not change with age. Rather, specific levels of mC and hmC differ with aging and by sex, depending on the chromosomal, promoter and CGI context. From a technical standpoint, these experiments demonstrate the need for appropriate quantitation controls, such as species-specific methylation standards, spike-in conversion controls, assessment of assay quantitative accuracy, differential quantitation of mC and hmC, and separation of CG and CH dinucleotide contexts.

### DNMT and TET regulation

Analysis of DNMT and TET expression with aging in males and females across independent sets of animals found no consistent changes in gene expression. Previous reports of hippocampal DNMT and TET expression with aging have shown a mixture of results, and the findings here provide clear evidence that these genes are not regulated with aging in either males or females. Additionally, examination of the public human gene expression data finds no evidence for changes in expression with aging. Many of the previous reports of differential expression have been of very small magnitude changes or have used immunohistochemical methods not well suited for quantitation [8, 21]. Using multiple animal cohorts of male and female mice, our findings should aid in resolving this ambiguity in how the expression of these genes changes with age. The possibility remains that differential expression may occur with aging in very specific subpopulations of cells or in other CNS regions which could be investigated in future studies. The equivalent expression of DNMTs and TETs in males and females provides evidence that the sex differences in total mC and hmC levels for the X chromosome, and the different levels of autosomes and sex chromosomes, are the result of altered targeting and not expression of these regulating enzymes.

Expression of DNMT3a2 (the short isoform of DNMT3a), previously reported as a regulator of cognitive aging [8], was not initially detectable in any of the young, adult, or old animals. DNMT3a2 preferentially localizes to euchromatin, while DNMT3a1 is predominantly found in heterochromatin [38], suggesting differential expression with aging of DNMT3a2 could have important genome regulatory effects. Using a state-of-the-art digital PCR approach, we found that DNMT3a2 expression rapidly declines from E11 to PN1 and is present in amounts marginally above background in the mature hippocampus. Even at these low levels of expression, no differences were observed with age in either males or females. The likely explanation for the difference in our findings from previous results is that the previous report of 20–25 % suppression of DNMT3a2 (and DNMT3a1) with aging assumed 100 % reaction efficiency, greater than the 75–76 % observed here, likely leading to an overestimation of the differences between groups. Even though our findings contradict the concept of declining hippocampal DNMT3a2 expression with aging, the improved learning and memory performance achieved by genetic induction of DNMT3a2 levels [8, 57] may be valid. However, a more accurate interpretation would be that of re-introduction of a developmentally silenced gene rather than restoration of an age-related decline in expression which leads to improved learning and memory.

### Genomic hypomethylation

The genomic hypomethylation hypothesis was first developed 40 years ago [58], and while many studies have examined methylation levels in blood samples from humans, this hypothesis has been extended to the CNS, being reported as generally accepted [15, 18–20]. However, for the brain and in this case the hippocampus in particular, the primary data from controlled laboratory animal models are divergent (Additional file 1: Table S1) and a hypomethylated genome with aging could also be expected to lead to a widespread increase in gene expression, which is not observed (e.g., [59]). Using antibody-based ELISAs of total genomic mC and bisulfite pyrosequencing of repeat elements, we find no hypomethylation with age. By the definitive approach of whole-genome oxBS-Seq, we also demonstrate that there is no evidence of genomic hypomethylation with age in the mouse hippocampus. Stated alternatively, the average levels of mC across the entire genome do not change with aging in either males or females.

Most of the previous reports of changes in total genomic methylation with aging have used antibody detection methods (ELISA and IHC). The technical limitations of these antibody-based methods are as follows: (1) they do not differentiate based on cytosine context (CG vs CH); (2) they are usually performed with methylation standards that have higher CG densities than found in mammalian genomes; and (3) the likelihood that antibody binding is biased against CG dense regions [60] reduces the reliability of these antibody-based measurements. Inclusion of appropriate methylation standards improved quantitation, but ultimately sequencing-based approaches are needed to quantify mC accurately and provide a genomic context to these data. The technical reasons for why previous reports have observed significant hypomethylation with aging are not as clear. This study specifically examined the hippocampus and analysis of other brain regions, tissues, and specific cell types with quantitatively rigorous techniques such as oxBS-Seq is needed. Future cell type-specific analyses of sorted or laser-captured selected cells are needed to provide cellular specificity to the understanding of changes in DNA methylation with aging. The agreement of our hippocampal data in terms of total genomic levels and patterns at specific genomic elements with the few datasets available from other brain regions supports the hypothesis that no hypomethylation with aging will be observed in other brain regions.

Our findings do provide evidence of some subtle differences in methylation by sex in sex chromosomes, as would be expected. Additionally, small age-related differences were observed in promoter regions when specifically analyzed. With the genome-wide approach used here, determination of the individual differentially methylated promoters (or CGIs) is not possible. However, in-depth oxBS analysis with base- and strand-specific mCG/mCH quantitation at appropriate sequencing depths may reveal subsets of CGIs, promoters, and other genomic regions (e.g., enhancers) that are differentially methylated with aging, sex, or both. In addition, the data presented here provide novel insights into methylation patterns in the brain. The specific hippocampal quantitation of mC confirms total mCG levels of ~50 % and mCH levels of <1 %. These values are lower than observed in bisulfite sequencing studies that combine mC and hmC, but are in agreement if the sum of our mC and hmC data is taken together [41]. Across CGIs and promoter regions, a U-shaped pattern was observed that correlates with previous findings from other brain regions [56]. The highly different mC levels within relatively small genomic regions of a few hundred base pairs further emphasize the need for base-specific quantitation. Alternative approaches to mC quantitation such as fragmentation and immunoprecipitation, which quantify over a region, and microarray approaches, which use a single CG site for a region, cannot resolve these patterns.

### Genomic hydroxymethylation

Sequencing data on hmC in the brain are quite limited since advanced techniques have only recently been developed and optimized [53, 54]. Utilizing the ability of oxBS-Seq to specifically quantify hmC, a total level of ~15 % hmCG was observed, which is in agreement with recent reports on human cortex and cerebellum [56, 61]. Sex chromosome hmCG levels are lower than autosomes. Intriguingly, the levels of hmCH are at an extremely low level, <0.01 %, similar to a previous report using an alternate sequencing method (TAB-Seq) [45]. While the ratio of mCG/hmCG is ~3, the ratio for mCH/hmCH is ~10, which may be a result of the higher affinity of TET enzymes for the CG over CH context [62].

Existing data on hippocampal hmC differences with aging or by sex are limited. Although we find that total hmC levels do not change with age, the higher level of hmCG in males than in females for the X chromosome has not been previously reported. Female samples did have more sequencing counts as compared to males as would be expected from having twice the X chromosomes, but this sex difference warrants further investigation. The age-related differences in hmCG in CGI shores and shelves were the inverse of those observed for mCG. This shift in males from mCG to hmCG in CGI shores and shelves with aging is a novel finding. Again, while these changes are relatively small in magnitude, more in-depth sequencing studies are needed to quantify differential methylation and hydroxymethylation at higher resolution with aging.

### Technical notes

We are unaware of other studies that have applied multiple different analytical techniques to examine mC and hmC dynamics with aging. The comparison of the results from these different approaches provides valuable insight into the technical performance of assays used in epigenomic studies. For ELISA-based quantitation, use of methylation standards with highly different CG density than the genome examined impairs quantitative accuracy and the absolute values differed from those observed by the more accurate sequencing approaches. Use of species-specific standards in the ELISA returned more accurate quantitation and avoided issues such as those in the literature where the sum of mC and hmC levels exceeds 100 % [24]. Nonetheless, the inability of ELISAs to differentiate between CG and CH contexts and quantitative limitations makes these widely reported measurements of limited value. It is not clear why some reports describe large differences in total genomic methylation with aging, but by the more accurate sequencing methods, which include conversion rate controls, presented here there is no evidence of changes in total methylation or hydroxymethylation levels. Pyrosequencing of repeat elements of the genome is often described as a surrogate for whole-genome levels [48, 49], though this was not found to be the case in our study. mC levels in LINE and SINE repeat elements approached 100 % rather than the ~50 % observed genome wide with oxidative bisulfite sequencing. The inclusion of synthetic spike-in controls in bisulfite and oxidation reactions provides a firmer quantitative foundation for sequencing studies. Previous approaches of using methylation in the CH context as the conversion control can overestimate failed conversion due to the natural occurrence of mCH. Finally, as demonstrated in the DNMT3a2 experiments, insufficient characterization of assays, e.g., determining reaction efficiencies, can apparently cause spurious findings of differences in gene expression.

## Conclusion

While the hypothesis of down-regulated DNMT and TET expression with aging and subsequent decreases in total levels of mC and/or hmC is seductive, the findings presented here provide extensive evidence that this is not the case in the murine hippocampus. Rather, these findings and others demonstrate the need to examine absolute methylation and hydroxymethylation levels across the genome in a base- and strand-specific manner with aging. In addition, our data point to the need to measure DNA modifications in both males and females, i.e., one cannot assume that age-related changes in DNA modifications in one sex will be mirrored in the other sex. Discovery of specific genomic loci with age-related changes in DNA modifications will enable studies of both the functional effects of an altered epigenome and the regulatory mechanisms that target changes to specific regions of the genome.

## Methods

### Animals

All animal experiments were performed according to protocols approved by the Penn State University and University of Oklahoma Health Sciences Center (OUHSC) Institutional Animal Care and Use Committees. Male and female C57BL/6 mice ages 3, 12, and 24 months were purchased from the National Institute on Aging Colony at Charles River Laboratories (Wilmington, MA). Set 1 mice were housed in the specific pathogen-free Pennsylvania State University College of Medicine Hershey Center for Applied Research facility in ventilated HEPA-filtered cages with ad libitum access to sterile chow (Harlan 2918 irradiated diet, Indianapolis, IN) and water. Set 2 mice were housed in the specific pathogen-free Rodent Barrier Facility at OUHSC in filter-top cages with ad libitum access to sterile chow (5053 Pico Lab; Purina Mills, St. Louis, MO) and water. In both facilities, all animals are free of helicobacter and parvovirus. For both sets of animals, following a one-week acclimation period on entering the respective facility, male mice were euthanized by decapitation. Female mice were euthanized during diestrus after estrous cycle staging.

Estrous cycle staging was performed by daily vaginal lavage to control for cycling differences. Lavages were conducted as described previously [55] and using well-established methods [63]. Briefly, sterile filtered water was expelled and aspirated approximately 4–5 times into the vaginal canal until enough cells were obtained for cytological analysis. Water from the vaginal wash was then placed onto a glass slide, allowed to dry, and then stained using 0.1 % crystal violet. The estrous cycle consists of three major phases: proestrus (high estrogen), estrus (low estrogen), and diestrus (low estrogen). Proestrus is defined by having a predominance of round, nucleated epithelial cells, estrus by cornified squamous epithelial cells, and diestrus by leukocytes with few epithelial cells present [63].

### Hippocampal cultures

Mixed co-cultures of neurons (both cortical and hippocampal) were isolated from C57BL/6 mice on embryonic day 18–20 (E18–20) in accordance with the approved Institutional Animal Care and Use Committees guidelines at OUHSC. Neurons were isolated as previously described [64]. In brief, following enzymatic digestion and trituration, hippocampal cells were resuspended in growth media [DMEM containing 2 % NuSerum, 10 % fetal bovine serum, penicillin (10 units/ml), streptomycin (10 μg/ml), and l-glutamine (29.2 μg/ml)] at a density of 300,000 cells/plate and seeded on 50 μg/ml poly-d-lysine-coated 6-cm dishes. Cells were fed every 3–4 days, with half of the conditioned media being replaced with fresh growth media. Neurons were treated with 30 µM fluorodeoxyuridine (FDU) on day 3 to reduce astrocyte proliferation. On day 9, neurons in culture were treated with either 50 µM of bicuculline (Sigma, St. Louis, MO) in vehicle (DMSO) or 50 mM of KCl for 4 h. Controls were either left untreated or treated with vehicle. Following treatment, neurons were rinsed with 0.1 M PBS pH 7.4 and harvested for further analysis.

### Quantitative PCR

RNA preparation was performed according to standard methods [AllPrep DNA/RNA Mini (Qiagen, Valencia, CA)] as described previously [65]. RNA quality was assessed by RNA 6000 Nano LabChip with an Agilent 2100 Expert Bioanalyzer (Agilent, Palo Alto, CA). Only samples with RNA integrity numbers greater than 7 were used in subsequent studies. RNA concentration was assessed by relative fluorescence using the RiboGreen assay (Invitrogen, Carlsbad, CA, USA).

cDNA was reverse-transcribed with random primers [ABI high-capacity cDNA reverse transcription kit (Life Technologies, Foster City, CA)] as previously described [66]. qPCR was performed with gene-specific primers and hydrolysis fluorogenic probes (Additional file 1: Table S5) (TaqMan, Life Technologies, and PrimeTime, IDT, Coralville, IA) as previously described [59]. Relative gene expression was calculated with ExpressionSuite version 1.0.3 software using the 2^−ΔΔ^Ct analysis method with β-actin as an endogenous control. β-actin was determined as a stable endogenous control through digital PCR across ages and sexes examined.

qPCR efficiency for each of the two DNMT3a2 assays was determined as follows: cDNA dilution series (1000, 750, 500, 250, 125 ng) was generated from mouse E13 brain RNA. qPCR was performed as above, the Ct was graphed against log (DNA copy #), and the slope of the line was used to determine efficiency [*E* = −1 + 10^(slope/−1)^] [67].

### Digital PCR

Digital PCR (dPCR) was carried out by mixing 3.33 μl diluted template DNA (50 ng/μl, diluted 1:10 for E11 and E18, 1:2 for PN1 and undiluted for 3 M hippocampus); concentrations of DNA are described below and varied by experiment with 16.5 μl QuantStudio 3D master mix, 3.33 μl 20× gene expression assay, and 9 μl water [32.16 μl (enough for two chips with excess)] (Life Technologies). Reactions were loaded onto QuantStudio 3D digital PCR chips (20,000 wells per chip) using the QuantStudio 3D chip loader according to manufacturer’s instructions (Life Technologies). Chips were then sealed and cycled on a GeneAmp PCR system 9700 with a flatblock attachment using the following conditions: Stage 1—96 °C for 10 min, Stage 2—60 °C for 2 min and then 98 °C for 30 s (repeat Stage 2 39 times), Stage 3—60 °C for 2 min and an infinite 10 °C hold. Chips were then read in the QuantStudio 3D chip reader (Life Technologies) to obtain raw fluorescent values per well. Quality check of the chips and counting of positive and negative wells in order to determine copies/microliter were carried out on the QuantStudio 3D AnalysisSuite cloud software (LifeTechnologies) using the absolute quantification module and then calculated to copies per μg of input RNA including incorporating dilutions stated above.

### Immunoblotting

Tissue (hippocampus or cortex as noted) or cell culture lysates were solubilized in a detergent-based protein lysis buffer containing protease and phosphatase inhibitors (100 mM NaCl, 20 mM HEPES, 1 mM EDTA, 1 mM dithiothreitol, 1.0 % Tween 20, 1 mM Na_3_VO_4_, 1 complete mini EDTA-free protease inhibitor cocktail tablet (Roche Applied Science, Indianapolis, IN, USA) for every 10 ml lysis buffer) using a bead mill (Retsch TissueLyzer II; Qiagen). Homogenates were incubated at 4 °C with gentle rocking for 15 min, and insoluble protein was removed by centrifugation (10,000*g*, 15 min, 4 °C). The soluble protein-containing supernatant was collected, and protein concentrations were determined by bicinchoninic acid (BCA) quantitation (Thermo Fisher, Waltham MA).

The DNMT3a1 protein standard was generated by transient transfection of a pCMV6 vector containing the full-length human DNMT3a1 (NM_175629, OriGene, Rockville MD) with a C-terminal Myc-DDK (FLAG) tag into HEK293T cells and cultured for 48 h. Cells were lysed with RIPA buffer [25 mM Tris–HCl pH7.6, 150 mM NaCl, 1 % NP-40, 1 mM EDTA, 1× proteinase inhibitor cocktail mix (Sigma), 1 mM PMSF and 1 mM Na_3_VO_4_] and protein concentrations determined by BCA assay. Protein was then affinity purified by anti-DDK (FLAG) antibody. Affinity-purified DNMT3a1-FLAG was loaded at 0.1 μg for testing detection by primary antibodies.

Immunoblotting was performed according to standard methods [68, 69]. Protein samples were adjusted to a concentration of 3 μg/μl in protein lysis buffer and LDS sample buffer (Invitrogen, Carlsbad, CA). Fifteen μg of each prepared protein sample (except where noted) was denatured at 95 °C and reduced with DTT prior to sodium dodecyl sulfate-polyacrylamide gel electrophoresis separation using criterion Tris–HCl precast 4–20 % acrylamide gradient gels (Bio-Rad, Hercules, CA, USA). An independent gel containing parallel aliquots of study samples was stained with deep purple total protein stain (GE Healthcare, Piscataway, NJ, USA) and quantitated by whole-lane digital densitometry (ImageQuant TL; Molecular Dynamics, Sunnyvale, CA, USA) to ensure equal protein content between samples as reported previously [68, 69]. For immunoblotting, proteins were transferred to polyvinylidene difluoride membranes (HyBond; GE Healthcare), blocked with 3 % BSA in PBS containing 1 % Tween-20, and incubated with primary antibodies (Additional file 1: Table S6). Membranes were washed with PBS containing 1 % Tween-20, incubated with species-appropriate secondary antibodies (Additional file 1: Table S6), and visualized with enhanced chemiluminescence substrate (GE Healthcare). Immunoreactive bands were imaged on film, digitized at a resolution of 800 d.p.i. with a transmissive scanner, and quantitated using automated digital densitometry software with rolling ball background subtraction (ImageQuant TL).

### mC and hmC ELISA

Total levels of mC and hmC, regardless of genomic context, were analyzed by colorimetric ELISAs (Zymo Research, D5326 and D5426, respectively, Irvine, CA). Isolated genomic DNA (as described above) concentrations were quantified by fluorescent assay (PicoGreen, Invitrogen) and all samples brought to the same concentration. ELISAs were performed in technical triplicates with 100 ng per well for mC and 200 ng for hmC after determining these amounts were within the linear range of the assays. For the first mC ELISA, Set 1 samples, the supplied 897-bp in vitro generated methylation standards were used. 5 % of these sequences are cytosines in CG contexts, much higher than the ~1 % found in the mouse genome. For the second performance of the mC ELISA, using Set 2 samples, the methylation standard curve was generated with a serial mixture of mouse gDNA methylation standards (low ~5 % and high ~95 %, EpigenDx, Hopkinton, MA) to provide an accurate quantitation of methylation levels. Methylation standards were quantified by pyrosequencing and whole-genome sequencing as previously described [70]. It should be noted, however, that these mouse methylation standards are only methylated at CG motifs as the standards are generated by in vitro treatment with M. SssI. The hmC ELISA standard curve was generated with the same synthetic standard as above, but with every cytosine in a CG context hydroxymethylated. No whole mouse genome hmC standards currently exist. In both cases, the antibody used in these ELISAs is specific to mC or hmC but cannot determine genomic locations or differentiate between CG and CH contexts.

### Repeat element pyrosequencing

Methylation levels of repetitive elements were examined as a surrogate of genome-wide methylation changes. Five hundred ng of gDNA (*n* = 4/group) was bisulfite converted (EZ DNA methylation-lighting, Zymo Research) and PCR amplified using primers for LINE-1 and B1 elements (EpigenDx). Following PCR amplification, pyrosequencing was conducted by EpigenDx and methylation was analyzed in these selected regions.

### Oxidative bisulfite sequencing

Oxidative bisulfite sequencing (oxBS) was performed according to previously described methods [53, 54]. For oxidative bisulfite sequencing, two parallel sets of libraries are constructed. The first uses traditional bisulfite conversion in which all Cs are converted through deamination to Us and mC and hmC are protected from conversion. In the second, DNA is oxidized with KRuO_4_ and all hmC are converted to 5fC. Subsequent bisulfite treatment converts all Cs and 5fCs to Us. In this manner, a specific mC signal is identified and hmC levels are determined by subtracting oxBS signal from BS signal.

Specifically, 400 ng of gDNA (*n* = 3/group) was sheared to an average size of 800 bp by sonication (E220, Covaris). DNA sizing was confirmed by capillary electrophoresis chip (Bioanalyzer DNA 1000, Agilent). One 200 ng aliquot of each sample was oxidized and then bisulfite converted, while the other 200 ng was only bisulfite converted (TrueMethyl, Cambridge Epigenetix, Cambridge, UK). Libraries were then constructed from the converted DNAs by end finishing and addition of Illumina adapters and indexes. Libraries were sized by capillary chip (high-sensitivity DNA chip, Agilent) and quantified by qPCR (Kappa Biosystems, Wilmington, MA). Libraries were loaded at 12 ρM and then sequenced by 150 bp paired-end reads on an Illumina NextSeq. Conversion rates for oxidation and bisulfite treatment for each sample preparation were determined from spike-in controls with unmodified Cs, as well as specific bases with mC, and hmC. Conversion rates were >99 % for bisulfite and >97 % for oxidation with minimal variance between samples (Additional file 1: Figure S1). The full complement of sequencing data has been posted to the Sequencing Read Archive (data to be publically released on publication).

Paired-end reads were filtered for low quality using a *Q*-score ≥25 and a maximum of 1 ambiguous nucleotide. Adapter trimming was performed in CLC Genomics Workbench 8.5.1 using Illumina universal adapter sequences. An additional 3 bp was trimmed off the 3′ and 5′ end of each of the paired-end reads. All computations were done in Unix and R using custom scripts unless specified otherwise. Reads were mapped to the reference mouse genome (GRCm38/mm10) using Bismark Bisulfite Mapper version 0.14.4 [71]. Female samples were mapped to all autosomes and chrX. Male samples were mapped to autosomes, chrX, and a chrY not containing homologous X sequences in order to maximize unique mapping. Methylation calls were made for both BS and oxBS sequences using bismark methylation extractor. The output from oxBS yields the methylation value for mC, while the output from BS yields the methylation value for mC and hmC. Average hmC levels were calculated as the difference between these two readouts (BS-oxBS).

Using a tiling approach, average of non-overlapping 100-bp windows for promoters and 200-bp windows for annotated CGI (16,009, http://hgdownload.soe.ucsc.edu/goldenPath/mm10/database/), shores, and shelves was computed in order to identify mC and hmC levels differences in CG-rich locations. Promoters were defined as the TSS ± 2 kb flanking region of all protein-coding genes included in the RefSeq database obtained from the UCSC website. Shores and shelf were defined as ±4 kb from the CGI [72] and were calculated based on the position relative to annotated CGIs. This sequencing approach allows differentiation of mC from hmC methylation, and levels can be separated by chromosome and between CG and CH contexts.

### Human microarray meta-analysis

All human microarray experimental data were obtained from NCBI’s GEO database of publicly available microarray experiments and collapsed to genes by max mean using the probe–gene mappings provided by the AILUN database [73]. The SQLite GEOmetadb database containing descriptions of the experiments was downloaded, and the age and tissue of each sample was extracted using text mining. The following random-effects linear model was fit to the dataset using R’s lme4 package, where expression indicates the per-gene *Z*-score of log-expression across all samples:

$${\text{Expression}}\sim{\text{Age}} + (1|{\text{TissueID}}) + (1|{\text{ExperimentID}}) \, + (1|{\text{PlatformID}})$$

Tissue of origin, experiment, and microarray platform are controlled for using random effects in this model. Results (*p* value, *t* statistic, and linear model coefficient) for the age coefficient of this formula are presented.

### Statistics

Statistical analyses were performed with SigmaStat 3.5 (SyStat Software, San Jose, CA) unless otherwise stated. Gene expression (qPCR and dPCR) data were analyzed by two-way ANOVA with the factors of sex and age. For factors and interactions passing the critical threshold (*α* < 0.05), appropriate Student–Newman–Keuls (SNK) pairwise post hoc testing with *α* < 0.05 was performed. ELISA and pyrosequencing data were analyzed by the same two-way ANOVA approach. Whole-genome mC and hmC data were analyzed by two-way ANOVA on counts from the specific genomic region or element as described.

## Additional file

10.1186/s13072-016-0080-6 **Figure S1.** Bisulfite and oxidation conversion rates. **Table S1.** Summary of reports of hippocampal genomic mC levels with aging. **Table S2.** Summary of reports of hippocampal DNMT expression with aging. **Table S3.** Summary of reports of hippocampal genomic hmC levels with aging. **Table S4.** Summary of reports of hippocampal DNMT expression with aging. **Table S5.** Gene expression assays. **Table S6.** Primary and secondary antibodies. **Table S7.** General signature of age-related DNMT and TET expression across all available data. **Table S8.** mC and hmC counts by context, chromosome, and sample.

## Abbreviations

mC: methylcytosine
hmC: hydroxymethylcytosine
DNMT: DNA methyltransferase
TET: ten-eleven translocation methylcytosine dioxygenase
fC: formylcytosine
caC: carboxylcytosine
CGI: CG island
mCG: methylated cytosine in the CG context
hmCG: hydroxymethylated cytosine in the CG context
mCH: methylated cytosine in the non-CG context
hmCH: hydroxymethylated cytosine in the non-CG context
BHMTC: Benjamini–Hochberg multiple testing correction
SNK: Student–Newman–Keuls

## References

[1] Lopez-Otin C, Blasco MA, Partridge L, Serrano M, Kroemer G (2013). The hallmarks of aging. Cell.

[2] Bocklandt S, Lin W, Sehl ME, Sanchez FJ, Sinsheimer JS, Horvath S (2011). Epigenetic predictor of age. PLoS ONE.

[3] Horvath S (2013). DNA methylation age of human tissues and cell types. Genome Biol.

[4] Marioni RE, Shah S, McRae AF, Chen BH, Colicino E, Harris SE (2015). DNA methylation age of blood predicts all-cause mortality in later life. Genome Biol.

[5] Miller CA, Sweatt JD (2007). Covalent modification of DNA regulates memory formation. Neuron.

[6] Rudenko A, Dawlaty MM, Seo J, Cheng AW, Meng J, Le T (2013). Tet1 is critical for neuronal activity-regulated gene expression and memory extinction. Neuron.

[7] Lister R, Mukamel EA (2015). Turning over DNA methylation in the mind. Front Neurosci.

[8] Oliveira AM, Hemstedt TJ, Bading H (2012). Rescue of aging-associated decline in Dnmt3a2 expression restores cognitive abilities. Nat Neurosci.

[9] Penner MR, Roth TL, Barnes CA, Sweatt JD (2010). An epigenetic hypothesis of aging-related cognitive dysfunction. Front Aging Neurosci.

[10] Lardenoije R, Iatrou A, Kenis G, Kompotis K, Steinbusch HW, Mastroeni D (2015). The epigenetics of aging and neurodegeneration. Prog Neurobiol.

[11] Kennedy BK, Berger SL, Brunet A, Campisi J, Cuervo AM, Epel ES (2014). Geroscience: linking aging to chronic disease. Cell.

[12] Kinde B, Gabel HW, Gilbert CS, Griffith EC, Greenberg ME (2015). Reading the unique DNA methylation landscape of the brain: non-CpG methylation, hydroxymethylation, and MeCP2. Proc Natl Acad Sci USA.

[13] Okano M, Bell DW, Haber DA, Li E (1999). DNA methyltransferases Dnmt3a and Dnmt3b are essential for de novo methylation and mammalian development. Cell.

[14] He YF, Li BZ, Li Z, Liu P, Wang Y, Tang Q (2011). Tet-mediated formation of 5-carboxylcytosine and its excision by TDG in mammalian DNA. Science.

[15] Pogribny IP, Vanyushin BF, Tollefsbol TO (2010). Age-Related Genomic Hypomethylation. Epigenetics of aging.

[16] Maegawa S, Hinkal G, Kim HS, Shen L, Zhang L, Zhang J (2010). Widespread and tissue specific age-related DNA methylation changes in mice. Genome Res.

[17] Martin GM (2009). Epigenetic gambling and epigenetic drift as an antagonistic pleiotropic mechanism of aging. Aging Cell.

[18] Zampieri M, Ciccarone F, Calabrese R, Franceschi C, Burkle A, Caiafa P (2015). Reconfiguration of DNA methylation in aging. Mech Ageing Dev.

[19] Chow HM, Herrup K (2015). Genomic integrity and the ageing brain. Nat Rev Neurosci.

[20] Irier HA, Jin P (2012). Dynamics of DNA methylation in aging and Alzheimer’s disease. DNA Cell Biol.

[21] Chouliaras L, van den Hove DL, Kenis G, Keitel S, Hof PR, van Os J (2012). Prevention of age-related changes in hippocampal levels of 5-methylcytidine by caloric restriction. Neurobiol Aging.

[22] Chen H, Dzitoyeva S, Manev H (2012). Effect of aging on 5-hydroxymethylcytosine in the mouse hippocampus. Restor Neurol Neurosci..

[23] Liu L, van Groen T, Kadish I, Li Y, Wang D, James SR (2011). Insufficient DNA methylation affects healthy aging and promotes age-related health problems. Clin Epigenetics..

[24] Mei Y, Jiang C, Wan Y, Lv J, Jia J, Wang X (2015). Aging-associated formaldehyde-induced norepinephrine deficiency contributes to age-related memory decline. Aging Cell.

[25] Tong Z, Han C, Qiang M, Wang W, Lv J, Zhang S (2015). Age-related formaldehyde interferes with DNA methyltransferase function, causing memory loss in Alzheimer’s disease. Neurobiol Aging.

[26] Singh P, Thakur MK (2014). Reduced recognition memory is correlated with decrease in DNA methyltransferase1 and increase in histone deacetylase2 protein expression in old male mice. Biogerontology.

[27] Elsner VR, Lovatel GA, Moyses F, Bertoldi K, Spindler C, Cechinel LR (2013). Exercise induces age-dependent changes on epigenetic parameters in rat hippocampus: a preliminary study. Exp Gerontol.

[28] Miller RA, Nadon NL (2000). Principles of animal use for gerontological research. J Gerontol A Biol Sci Med Sci.

[29] Szulwach KE, Li X, Li Y, Song CX, Wu H, Dai Q (2011). 5-hmC-mediated epigenetic dynamics during postnatal neurodevelopment and aging. Nat Neurosci.

[30] Chouliaras L, van den Hove DL, Kenis G, Keitel S, Hof PR, van Os J (2012). Age-related increase in levels of 5-hydroxymethylcytosine in mouse hippocampus is prevented by caloric restriction. Curr Alzheimer Res.

[31] Stilling RM, Benito E, Gertig M, Barth J, Capece V, Burkhardt S (2014). De-regulation of gene expression and alternative splicing affects distinct cellular pathways in the aging hippocampus. Front Cell Neurosci.

[32] McCarthy MM, Arnold AP, Ball GF, Blaustein JD, De Vries GJ (2012). Sex differences in the brain: the not so inconvenient truth. J Neurosci.

[33] Mielke MM, Vemuri P, Rocca WA (2014). Clinical epidemiology of Alzheimer’s disease: assessing sex and gender differences. Clin Epidemiol.

[34] Austad SN, Bartke A (2015). Sex differences in longevity and in responses to anti-aging interventions: a mini-review. Gerontology..

[35] Pankevich DE, Wizemann T, Altevogt BM (2011). Sex differences and implications for translational neuroscience research: workshop summary.

[36] Heard E, Clerc P, Avner P (1997). X-chromosome inactivation in mammals. Annu Rev Genet.

[37] Clayton JA, Collins FS (2014). Policy: NIH to balance sex in cell and animal studies. Nature.

[38] Chen T, Ueda Y, Xie S, Li E (2002). A novel Dnmt3a isoform produced from an alternative promoter localizes to euchromatin and its expression correlates with active de novo methylation. J Biol Chem.

[39] Weisenberger DJ, Velicescu M, Preciado-Lopez MA, Gonzales FA, Tsai YC, Liang G (2002). Identification and characterization of alternatively spliced variants of DNA methyltransferase 3a in mammalian cells. Gene.

[40] Feng J, Chang H, Li E, Fan G (2005). Dynamic expression of de novo DNA methyltransferases Dnmt3a and Dnmt3b in the central nervous system. J Neurosci Res.

[41] Guo JU, Su Y, Shin JH, Shin J, Li H, Xie B (2014). Distribution, recognition and regulation of non-CpG methylation in the adult mammalian brain. Nat Neurosci.

[42] Suetake I, Morimoto Y, Fuchikami T, Abe K, Tajima S (2006). Stimulation effect of Dnmt3L on the DNA methylation activity of Dnmt3a2. J Biochem.

[43] Sakai Y, Suetake I, Shinozaki F, Yamashina S, Tajima S (2004). Co-expression of de novo DNA methyltransferases Dnmt3a2 and Dnmt3L in gonocytes of mouse embryos. Gene Expr Patterns.

[44] Palamarchuk A, Yan PS, Zanesi N, Wang L, Rodrigues B, Murphy M (2012). Tcl1 protein functions as an inhibitor of de novo DNA methylation in B-cell chronic lymphocytic leukemia (CLL). Proc Natl Acad Sci USA.

[45] Lister R, Mukamel EA, Nery JR, Urich M, Puddifoot CA, Johnson ND (2013). Global epigenomic reconfiguration during mammalian brain development. Science.

[46] Chen L, Chen K, Lavery LA, Baker SA, Shaw CA, Li W (2015). MeCP2 binds to non-CG methylated DNA as neurons mature, influencing transcription and the timing of onset for Rett syndrome. Proc Natl Acad Sci USA.

[47] He Y, Ecker JR (2015). Non-CG Methylation in the Human Genome. Annu Rev Genomics Hum Genet.

[48] Yang AS, Estecio MR, Doshi K, Kondo Y, Tajara EH, Issa JP (2004). A simple method for estimating global DNA methylation using bisulfite PCR of repetitive DNA elements. Nucleic Acids Res.

[49] Weisenberger DJ, Campan M, Long TI, Kim M, Woods C, Fiala E (2005). Analysis of repetitive element DNA methylation by MethyLight. Nucleic Acids Res.

[50] Mouse Genome Sequencing C, Waterston RH, Lindblad-Toh K, Birney E, Rogers J, Abril JF (2002). Initial sequencing and comparative analysis of the mouse genome. Nature.

[51] Khare T, Pai S, Koncevicius K, Pal M, Kriukiene E, Liutkeviciute Z (2012). 5-hmC in the brain is abundant in synaptic genes and shows differences at the exon-intron boundary. Nat Struct Mol Biol.

[52] Yu M, Hon GC, Szulwach KE, Song CX, Zhang L, Kim A (2012). Base-resolution analysis of 5-hydroxymethylcytosine in the mammalian genome. Cell.

[53] Booth MJ, Branco MR, Ficz G, Oxley D, Krueger F, Reik W (2012). Quantitative sequencing of 5-methylcytosine and 5-hydroxymethylcytosine at single-base resolution. Science.

[54] Booth MJ, Ost TW, Beraldi D, Bell NM, Branco MR, Reik W (2013). Oxidative bisulfite sequencing of 5-methylcytosine and 5-hydroxymethylcytosine. Nat Protoc.

[55] Mangold CA, Masser DR, Stanford DR, Bixler GV, Pisupati A, Giles CB, et al. CNS-wide sexually dimorphic induction of the major histocompatibility complex 1 pathway with aging. J Gerontol A Biol Sci Med Sci. 2016:glv232 **[Epub ahead of print]** 10.1093/gerona/glv232 PMC5155655 26786204

[56] Wen L, Li X, Yan L, Tan Y, Li R, Zhao Y (2014). Whole-genome analysis of 5-hydroxymethylcytosine and 5-methylcytosine at base resolution in the human brain. Genome Biol.

[57] Oliveira AM, Hemstedt TJ, Freitag HE, Bading H. Dnmt3a2: a hub for enhancing cognitive functions. Mol Psychiatry. 2015:glv232 **[Epub ahead of print]** 10.1038/mp.2015.175 26598069

[58] Vanyushin BF, Nemirovsky LE, Klimenko VV, Vasiliev VK, Belozersky AN (1973). The 5-methylcytosine in DNA of rats. Tissue and age specificity and the changes induced by hydrocortisone and other agents. Gerontologia.

[59] Masser DR, Bixler GV, Brucklacher RM, Yan H, Giles CB, Wren JD (2014). Hippocampal subregions exhibit both distinct and shared transcriptomic responses to aging and nonneurodegenerative cognitive decline. J Gerontol A Biol Sci Med Sci.

[60] Nair SS, Coolen MW, Stirzaker C, Song JZ, Statham AL, Strbenac D (2011). Comparison of methyl-DNA immunoprecipitation (MeDIP) and methyl-CpG binding domain (MBD) protein capture for genome-wide DNA methylation analysis reveal CpG sequence coverage bias. Epigenetics.

[61] Lunnon K, Hannon E, Smith RG, Dempster E, Wong C, Burrage J (2016). Variation in 5-hydroxymethylcytosine across human cortex and cerebellum. Genome Biol.

[62] Hu L, Li Z, Cheng J, Rao Q, Gong W, Liu M (2013). Crystal structure of TET2-DNA complex: insight into TET-mediated 5mC oxidation. Cell.

[63] McLean AC, Valenzuela N, Fai S, Bennett SA. Performing vaginal lavage, crystal violet staining, and vaginal cytological evaluation for mouse estrous cycle staging identification. Journal of visualized experiments : JoVE. 2012;67:e4389. 10.3791/4389 PMC3490233 23007862

[64] Ashpole NM, Song W, Brustovetsky T, Engleman EA, Brustovetsky N, Cummins TR (2012). Calcium/calmodulin-dependent protein kinase II (CaMKII) inhibition induces neurotoxicity via dysregulation of glutamate/calcium signaling and hyperexcitability. J Biol Chem.

[65] Imperio CG, McFalls AJ, Colechio EM, Masser DR, Vrana KE, Grigson PS, et al. Assessment of individual differences in the rat nucleus accumbens transcriptome following taste-heroin extended access. Brain Res Bull. 2015:glv232 **[Epub ahead of print]** 10.1016/j.brainresbull.2015.12.005 PMC4893904 26733446

[66] Van Kirk CA, VanGuilder HD, Young M, Farley JA, Sonntag WE, Freeman WM (2011). Age-related alterations in retinal neurovascular and inflammatory transcripts. Mol Vis.

[67] Freeman WM, Walker SJ, Vrana KE. Quantitative RT-PCR: pitfalls and potential. Biotechniques. 1999;26:112–122, 124–115. 10.2144/99261rv01 9894600

[68] VanGuilder HD, Farley JA, Yan H, Van Kirk CA, Mitschelen M, Sonntag WE (2011). Hippocampal dysregulation of synaptic plasticity-associated proteins with age-related cognitive decline. Neurobiol Dis.

[69] VanGuilder HD, Yan H, Farley JA, Sonntag WE, Freeman WM (2010). Aging alters the expression of neurotransmission-regulating proteins in the hippocampal synaptoproteome. J Neurochem.

[70] Masser DR, Stanford DR, Hadad N, Giles CB, Wren JD, Sonntag WE (2016). Bisulfite oligonucleotide-capture sequencing for targeted base- and strand-specific absolute 5-methylcytosine quantitation. Age (Dordr).

[71] Krueger F, Andrews SR (2011). Bismark: a flexible aligner and methylation caller for Bisulfite-Seq applications. Bioinformatics.

[72] Doi A, Park IH, Wen B, Murakami P, Aryee MJ, Irizarry R (2009). Differential methylation of tissue- and cancer-specific CpG island shores distinguishes human induced pluripotent stem cells, embryonic stem cells and fibroblasts. Nat Genet.

[73] Chen R, Li L, Butte AJ (2007). AILUN: reannotating gene expression data automatically. Nat Methods.

