# Supplementary material for: Absence of genomic hypomethylation or regulation of cytosine-modifying enzymes with aging in male and female mice
Source: Epigenetics Chromatin. 2016 Jul 13;9:30. doi: 10.1186/s13072-016-0080-6 (PMC4942942; doi:10.1186/s13072-016-0080-6)
Supplement: Supplementary file 1 — 10.1186/s13072-016-0080-6 Figure S1. Bisulfite and oxidation conversion rates. Table S1. Summary of reports of hippocampal genomic mC levels with aging. Table S2. Summary of reports of hippocampal DNMT expression with aging. Table S3. Summary of reports of hippocampal genomic hmC levels with aging. Table S4. Summary of reports of hippocampal DNMT expression with aging. Table S5. Gene expression assays. Table S6. Primary and secondary antibodies. Table S7. General signature of age-related DNMT and TET expression across all available data. Table S8. mC and hmC counts by context, chromosome, and sample. [file 13072_2016_80_MOESM1_ESM.pdf]

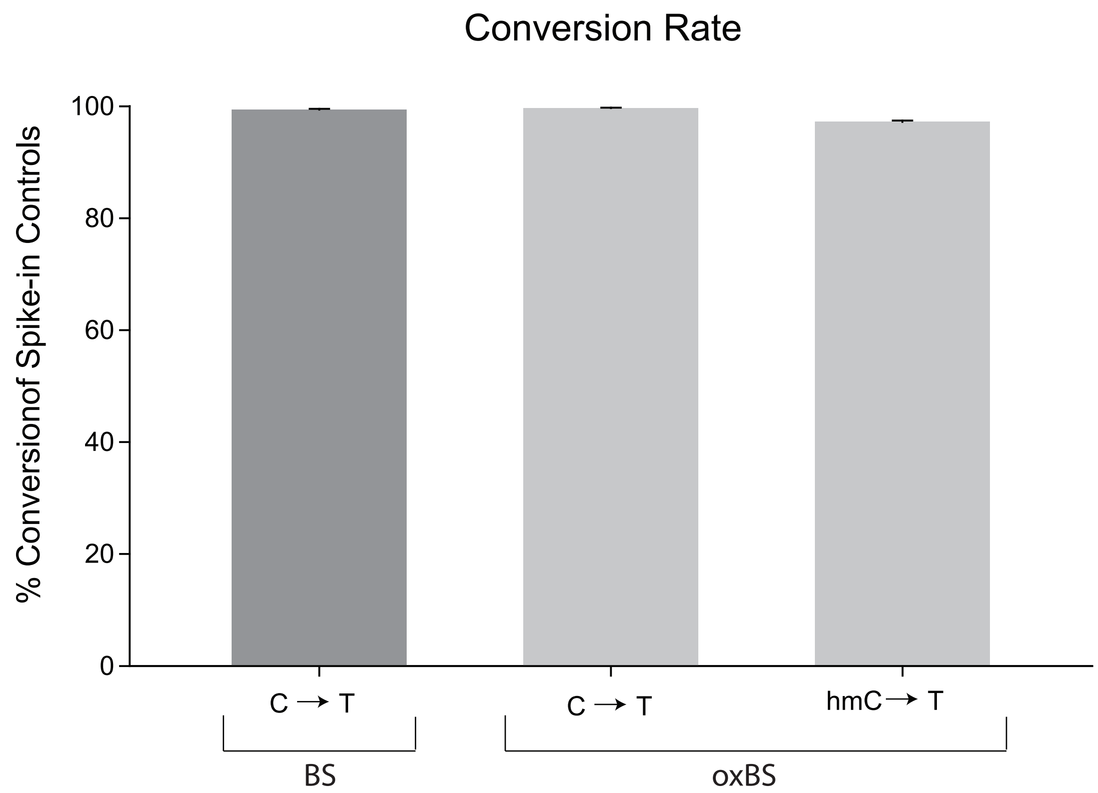

Figure S1: Bisulfite and oxidation conversion rates. For each sample spike-in control sequences with C, mC and hmC bases was used to determine conversion rates. Data are presented as mean +/- S.D. for all samples.

	Total genomic mC levels				
	Species	*Sex*	*Assay*	*Ages(Months)*	*Reference*
**Increase**	Mouse C57BL/6	Male	IHC	12, 24↑	(Chouliaras *et al.* 2012b)
**No Change**	Mouse C57BL/6	Male	ELISA	2, 22↔, 24↔	(Chen *et al.* 2012)
**Decreased**	Mouse C57BL/6	Male	LC	7, 13↓, 19↓	(Liu *et al.* 2011)
	Rat Sprague Dawley	Male	ELISA	2, 8↔, 16↓, 30↓	(Mei *et al.* 2015)
	Rat Sprague Dawley	Not Stated	ELISA	3, 16↓, 30↓	(Tong *et al.* 2015)

Table S1: Summary of reports of hippocampal genomic mC levels with aging. Species and strain are given along with the sex of animals used. Assay type and ages of animals are given. Arrows indicate increases, decrease, or no change for that age as compared to the youngest age indicated. IHC – immunohistochemistry, ELISA - enzyme-linked immunosorbent assay, LC – liquid chromatography.

	DNMT expression							
	Gene	*Species*	*Sex*	*mRNA*	*Protein*	*Assay*	*Ages(Months)*	*Reference*
**Increase**	DNMT3a (1/2)	Mouse C57BL/6	Male		X	IHC	12, 24↑	(Chouliaras *et al.* 2012b)
**No Change**	DNMT1	Mouse C57BL/6	Male	X		qPCR	3, 18↔	(Oliveira *et al.* 2012)
	DNMT1	Mouse Swiss Albino	Male	X		qPCR	2.5, 7.5↔, 20↔	(Singh & Thakur 2014)
	DNMT3a	Mouse Swiss Albino	Male	X		qPCR	2.5, 7.5↔, 20↔	(Singh & Thakur 2014)
	DNMT3a	Mouse Swiss Albino	Male		X	IB	2.5, 7.5↔, 20↔	(Singh & Thakur 2014)
	DNMT3b	Mouse Swiss Albino	Male	X		qPCR	2.5, 7.5↔, 20↔	(Singh & Thakur 2014)
	DNMT3b	Mouse C57BL/6	Male	X		qPCR	3, 18↔	(Oliveira *et al.* 2012)
	DNMT3b	Rat	Male		X	ELISA	3, 20↔	(Elsner *et al.* 2013)
	DNMT3b	Mouse Swiss Albino	Male		X	IB	2.5, 7.5↔, 20↔	(Singh & Thakur 2014)
**Decreased**	DNMT1	Rat	Male		X	ELISA	3, 20↓	(Elsner *et al.* 2013)
	DNMT1	Mouse Swiss Albino	Male		X	IB	2.5, 7.5↓ 20↓	(Singh & Thakur 2014)
	DNMT1	Rat Sprague Dawley	Not Stated		X	IB	3, 16↓, 30↓	(Tong *et al.* 2015)
	DNMT3a1/2	Rat Sprague Dawley	Not Stated		X	IB	3, 16↓, 30↓	(Tong *et al.* 2015)
	DNMT3a1	Mouse C57BL/6	Male	X		qPCR	3, 18↓	(Oliveira *et al.* 2012)
	DNMT3a2	Mouse C57BL/6	Male	X		qPCR	3, 18↓	(Oliveira *et al.* 2012)

Table S2: Summary of reports of hippocampal DNMT expression with aging. The genes, species and strain are given along with the sex of animals used. Assay type and endpoint (mRNA or protein) and ages of animals are given. Arrows indicate increases, decrease, or no change for that age as compared to the youngest age indicated. IB – immunoblotting, ELISA - enzyme-linked immunosorbent assay, qPCR – quantitative PCR.

	**Total genomic hmC levels**				
	Species	*Sex*	*Assay*	*Ages(Months)*	*Reference*
**Increase**	Mouse C57BL/6	Not Stated	Dot Blot	0.25, 1.5↑, 12↑	(Szulwach *et al.* 2011)
	Mouse C57BL/6	Male	ELISA	2, 22↑, 24↑	(Chen *et al.* 2012)
	Mouse C57BL/6	Male	IHC	12, 24↑	(Chouliaras *et al.* 2012a)

Table S3: Summary of reports of hippocampal genomic hmC levels with aging. Species and strain are given along with the sex of animals used. Assay type and ages of animals are given. Arrows indicate increases as compared to the youngest age indicated. No reports were found for decreases or lack of changes. IHC – immunohistochemistry, ELISA - enzyme-linked immunosorbent assay.

	TET expression							
	Gene	*Species*	*Sex*	*mRNA*	*Protein*	*Assay*	*Ages(Months)*	*Reference*
**Increase**	No reports							
**No Change**	TET1	Mouse C57BL/6	Male	X		qPCR	2, 22↔, 24↔	(Chen *et al.* 2012)
	TET2	Mouse C57BL/6	Male	X		qPCR	2, 22↔, 24↔	(Chen *et al.* 2012)
	TET3	Mouse C57BL/6	Male	X		qPCR	2, 22↔, 24↔	(Chen *et al.* 2012)
**Decreased**	Tet1	Mouse C57BL/6	Not Stated	X		microarray	3, 28-30↓	(Stilling *et al.* 2014)

Table S4: Summary of reports of hippocampal DNMT expression with aging. The genes, species and strain are given along with the sex of animals used. Assay type and endpoint (mRNA or protein) and ages of animals are given. Arrows indicate increase, decrease, or no change for that age as compared to the youngest age indicated. qPCR – quantitative PCR.

Gene	NCBI Gene ID	Gene Name	Assay # (Invitrogen)	Alias
DNMT1	13433	DNA methyltransferase 1	Mm01151063_m1	Cxxc9, Dnmto, MCMT, MTase, Met-1, Met1, MommeD2, m.MmuI, Dnmt1
DNMT3a (pan)	13435	DNA methyltransferase 3A	Mm00432881_m1	MmuIIIA
DNMT3a1	13435	DNA methyltransferase 3A isoform 1	Mm00432870_m1	MmuIIIA
DNMT3a2 #1	13435	DNA methyltransferase 3A isoform 2	Mm00463987_m1	MmuIIIA
DNMT3a2 #2	13435	DNA methyltransferase 3A isoform 2	*Custom Assay	MmuIIIA
DNMT3b	13436	DNA methyltransferase 3B	Mm01240113_m1	MmuIIIB
TET1	52463	tet methylcytosine dioxygenase 1	Mm01169087_m1	2510010B09Rik, AA517754, BB001228, Cxxc6, D10Ertd17e, LCX, mKIAA1676
TET2	214133	tet methylcytosine dioxygenase 2	Mm00524395_m1	Ayu17-449, E130014J05Rik, mKIAA1546
TET3	194388	tet methylcytosine dioxygenase 3	Mm00805756_m1	B430006D22Rik, BC037432, D230004J03Rik
DNMT3a2 #2	**Assay Name**	**reverse sequence**	**forward sequence**	**Probe**
	Nm153743.1.pt.Dnmt3a2	TGGTTCTCTTCCACAGCATTC	GACGGGCAGCTATTTACAGAG	/56-FAM/CTTCTCAGC/ZEN/CCATAAGGCCAGGT/3IABkFQ/

Table S5: Gene Expression Assays

Specificity	Immunogen	Host	Clonal	Supplier	Catalog
Dnmt3a1	AA 42-67 near N-term of human Dnmt3a	mouse	monoclonal	santa cruz	sc-365769
Dnmt3a1	AA 10-118 of human Dnmt3a	rabbit	polyclonal	thermo/pierce	PA3-16557
Dnmt3a1/ Dnmt3a2	AA 723-752 of C-term of human Dnmt3a/3a2	rabbit	polyclonal	thermo/pierce	PA5-11141
Dnmt3a1/ Dnmt3a2	AA 705-908 of C-term of mouse Dnmt3a (clone 64B1446)	mouse	monoclonal	thermo/pierce	MA5-16171
FLAG tag	Synthetic peptide (DYKDDDDK)	mouse	monoclonal	thermo/pierce	MA1-91878

Secondary Antibody	Host	Clonal	Supplier	Catalog
Rabbit Trueblot®: Anti-Rabbit IgG HRP	mouse	monoclonal	Rockland	18-8816-31
Mouse Trueblot®Ultra: Anti-Mouse IgG HRP	rat	monoclonal	Rockland	PA3-16557

Table S6: Primary and secondary antibodies.

Entrez ID	Symbol	Chr	Name	coef	SE	t	p-value
80312	TET1	10	tet methylcytosine dioxygenase 1	0.0010	0.006	0.1808	0.86
54790	TET2	4	tet methylcytosine dioxygenase 2	-0.0021	0.005	-0.4098	0.68
200424	TET3	2	tet methylcytosine dioxygenase 3	-0.0010	0.005	-0.1881	0.85
1786	DNMT1	19	DNA (cytosine-5-)-methyltransferase 1	-0.0077	0.007	-1.0773	0.28
1788	DNMT3A	2	DNA (cytosine-5-)-methyltransferase 3 alpha	-0.0044	0.004	-1.0994	0.27
1789	DNMT3B	20	DNA (cytosine-5-)-methyltransferase 3 beta	-0.0006	0.004	-0.1485	0.88

Table S7: General signature of age-related DNMT and TET expression across all available data. To test whether the expression of these genes changes significantly with age, controlling for tissue and experimental platform, we used a linear mixed effects (lme4) model: (GeneExpression ~ Age + (1 | TissueID) + (1 | ExperimentID) + (1 | PlatformID)). Shown above is the age term. “Coef” describes the estimated coefficient of the change in z-score per year for the expression of each gene, the standard error (SE) and t-statistic for the analysis.

CG

Bisulfite Library (BS)

	sample	Counts_Autosomes	Counts_ ChrX	Counts_chrY	Total
Female Aged	1	24,694,355	844,641		
	2	21,169,401	686,924		
	3	20,023,913	673,221		68,092,455
Female Young	1	17,735,233	528,418		
	2	16,111,724	415,782		
	3	27,425,650	767,989		62,984,796
Male Young	1	15,742,745	237,236	27,078	
	2	16,164,827	239,198	27,411	
	3	20,800,830	344,693	44,449	53,628,467
Male Aged	1	17,873,252	273,735	31,271	
	2	15,591,021	231,093	28,560	
	3	25,102,455	417,266	53,867	59,602,520

Oxidized - Bisulfite Library (oxBS)

	sample	Counts_Autosomes	Counts_ ChrX	Counts_chrY	Total
Female Aged	1	19,385,928	674,049		
	2	23,698,637	780,465		
	3	26,752,478	911,294		72,202,851
Female Young	1	27,910,345	852,679		
	2	14,446,412	403,103		
	3	24,976,106	697,517		69,286,162
Male Young	1	19,417,441	311,846	37,886	
	2	15,363,729	235,046	27,070	
	3	12,861,033	207,711	26,884	48,488,646
Male Aged	1	13,106,521	212,874	26,064	
	2	15,454,120	244,804	30,911	
	3	11,446,059	195,233	24,727	40,741,313

CH

Bisulfite Library (BS)

	sample	Counts_Autosom	Counts_ ChrX	Counts_chrY	Total
Female Aged	1	315,640,364	356,420,253		
	2	267,156,912	299,200,906		
	3	262,103,810	295,423,446		1,795,945,691
Female Young	1	226,500,152	250,883,392		
	2	182,636,568	199,178,286		
	3	346,340,017	384,165,963		1,589,704,378
Male Young	1	181,069,600	191,957,079	888,276	
	2	191,984,671	202,846,537	911,586	
	3	265,080,607	284,726,583	1,522,105	1,320,987,044
Male Aged	1	209,339,181	222,746,040	1,033,079	
	2	175,076,197	184,911,850	873,371	
	3	313,821,904	337,046,872	1,820,030	1,446,668,524

Oxidized - Bisulfite Library (oxBS)

	sample	Counts_Autosom	Counts_ ChrX	Counts_chrY	Total
Female Aged	1	245,938,321	278,518,521		
	2	284,943,870	319,092,273		
	3	344,530,585	389,172,131		1,862,195,701
Female Young	1	345,064,532	383,233,204		
	2	169,866,676	187,359,530		
	3	308,607,694	340,980,759		1,735,112,395
Male Young	1	233,964,528	249,365,544	1,279,626	
	2	173,979,958	183,838,636	857,548	
	3	158,991,021	170,007,603	906,921	1,173,191,385
Male Aged	1	153,868,737	164,062,765	857,764	
	2	185,047,852	197,339,345	1,048,238	
	3	133,463,340	142,865,197	788,222	979,341,460

Table S8: mC and hmC counts by context, chromosome and sample. In the oxBS workflow two library are made for each sample. The first is bisulfite converted and the second is oxidized and bisulfite converted. The counts presented here are total the number of cytosines read , whether read as C or T, by chromosomal and CG/CH context. mC levels are determined from the oxBS library and hmC levels are determined by BS - oxBS.
